# Near-Complete Genome Sequence of a Fish Nervous Necrosis Virus Isolated from a Clinical Disease Outbreak in Farm-Reared Bream *Sparus aurata* in Spain

**DOI:** 10.1128/genomeA.01392-17

**Published:** 2018-01-04

**Authors:** Panagiota Stathopoulou, Nausika Rafailidou, Kostas Tzokas, Costas Batargias, George Tsiamis

**Affiliations:** a Department of Environmental and Natural Resources Management, University of Patras, Agrinio, Greece; b Andromeda S.A., Agios Vasilios, Rion, Greece; c Department of Fisheries & Aquaculture Management, Technological Educational Institute of Western Greece, Messolonghi, Greece

## Abstract

*Sparus aurata* larvae infected by viral nervous necrosis were collected from an aquaculture fish farm. The isolated viral genome, composed of two segments (RNA1 and RNA2), was sequenced and analyzed comparatively. Phylogenetic tree analyses revealed that the isolated strain is a reassortant, exhibiting a red-spotted grouper nervous necrosis virus (RGNNV)-type RNA1 and a striped jack nervous necrosis virus (SJNNV)-type RNA2.

## GENOME ANNOUNCEMENT

Viral nervous necrosis (VNN) is a disease observed in marine aquaculture hatcheries all over the world (1). Larval or juvenile stages are more prone to this viral infection, which results in high mortality rates, but significant mortality can also occur in older fish up to production size (2). Currently, more than 50 fish species have been reported to be affected by VNN (1, 3). VNN is caused by nervous necrosis viruses (NNV), which belong to the genus *Betanodavirus*, family *Nodaviridae*. NNVs are single-stranded positive-sense RNA viruses composed of two segments. RNA1 is the largest segment, approximately 3.1 kb in length, and encodes the RNA-dependent RNA polymerase (RdRp) (4, 5). The second segment, RNA2, is about 1.4 kb in length and encodes a precursor to the coat protein (CP), the single structural component of the NNV virion (6). Nodaviruses are categorized into 4 different genotypes based on a specific region (T4) of the CP sequence (427 bp), barfin flounder nervous necrosis virus (BFNNV), red-spotted grouper nervous necrosis virus (RGNNV), striped jack nervous necrosis virus (SJNNV), and tiger puffer nervous necrosis virus (TPNNV) (7).

*Sparus aurata* larvae were collected from an aquaculture fish farm in Spain based on their clinical signs and molecular virus detection. First, larvae homogenization was followed by RNA extraction and first-strand cDNA synthesis. cDNA was used as template in all reactions. PCR was used to amplify overlapping fragments in the middle of both the RNA1 and RNA2 genes by using different primers, which were designed on the basis of known betanodaviruses. Then, PCR products were subcloned into a pGEM-T easy vector for extended sequencing read lengths. Phylogenetic analysis was conducted with MEGA software v.6 (8) using the neighbor-joining method (1,000 bootstrap replicates) and the Jukes-Cantor model.

Following assembly of the data from overlapping fragments, the near-complete genome was composed of two open reading frames (ORFs) of 2,826 bases (RNA1) and 1,367 bases (RNA2). More specifically, the RNA1 contains an open reading frame (ORF) encoding the near-complete RdRp, which is composed of 942 amino acids and presented 95% identity to known RdRps. The RNA2 contains a complete ORF encoding the CP, which is composed of 340 amino acids and revealed 97% highest identity to known CPs.

Comparative analysis at the nucleotide level showed that the RNA1 isolate sequence was 98% identical to other reference sequences and grouped with RGNNV genotypes. In contrast, the RNA2 isolate sequence presented 98% homology with SJNNV genotypes. Phylogenetic tree analysis revealed that *Sparus aurata* betanodavirus isolate SpSaBI 2015 is a reassortant, exhibiting an RGNNV-type RNA1 and an SJNNV-type RNA2. Although there have been many reports in southern Europe of natural reassortant viruses in the form of RGNNV/SJNNV, harboring the RNA1 of the RGNNV and the RNA2 of the SJNNV, and in the form of SJNNV/RGNNV, harboring the RNA1 of the SJNNV and the RNA2 of the RGNNV (9–11), the role of genetic reassortment in viral phenotypes and its effect on biological and ecological properties of the viruses is still unexplored.

### Accession number(s).

The gene sequences have been deposited in GenBank under the accession numbers KY785169 (RNA2) and KY785170 (RNA1).
